# Overexpression of interleukin‐10 in engineered macrophages protects endothelial cells against LPS‐induced injury *in vitro*

**DOI:** 10.1002/2211-5463.13365

**Published:** 2022-01-26

**Authors:** Lingxian Yi, Tujun Weng, Penghui Nie, Lin Zhu, Mingming Gao, Hongxing Jia, Shaohua Yang, Xiubin Li, Luo Zhang, Ye Xu, Penglin Ma, Mei Hu

**Affiliations:** ^1^ Department of Critical Care Medicine Strategic Support Force Medical Center Beijing China; ^2^ Chinese PLA Medical School Chinese PLA General Hospital Beijing China; ^3^ Senior Department of Orthopaedics The Fourth Medical Centre Chinese PLA General Hospital Beijing China; ^4^ School of Mechanical Engineering and Automation Beihang University Beijing China; ^5^ Beijing Key Laboratory of Organ Transplantation and Immunology Regulatory The 8th Medical Center Chinese PLA General Hospital Beijing China; ^6^ Department of Biomedical Engineering The Fifth Medical Center Chinese PLA General Hospital Beijing China; ^7^ Critical Care Medicine Department Guiqian International General Hospital Guiyang China

**Keywords:** endothelial permeability, engineered macrophages, noncontact coculture, sepsis, vascular endothelial cell

## Abstract

Endothelial dysfunction is a primary pathophysiological change in sepsis. Macrophages are known to interact with vascular endothelial cells during the development of sepsis. Recently, drug delivery based on engineered macrophages was reported as an alternative approach for the management of diseases. Interleukin‐10 (IL10) is a well‐known anti‐inflammatory cytokine, which reduces inflammation and inhibits dysfunction of endothelial cells caused by sepsis. It is currently poorly understood whether genetically modified macrophages with overexpression of IL10 are able to restore endothelial integrity and function at the cellular level. In this study, we used lentiviral vectors to construct RAW264.7 macrophages engineered to overexpress IL10 (IL10‐eM) and investigated the effects of the IL10‐eM supernatant on LPS‐induced endothelial dysfunction using a noncontact coculture system. We found that cotreatment with IL10‐eM supernatant significantly attenuates the effects of LPS‐induced dysfunction of endothelial cells, including endothelial inflammatory response, endothelial permeability, and apoptosis. In addition, we discovered that LPS‐induced downregulation of VE‐cadherin and high production of reactive oxygen species were significantly attenuated upon IL10‐eM exposure. Furthermore, upregulation of IL6, TNFα, and Bax was decreased after treatment of cells with IL10‐eM supernatant. These results demonstrated that supernatant from engineered macrophages genetically modified with IL10 can effectively protect endothelial cells against LPS‐induced dysfunction *in vitro*, suggesting that exosomes from such engineered macrophages may have therapeutic effects against sepsis.

## Graphical abstract

Restoration of vascular endothelial cells (VECs) dysfunction might be a potential therapeutic strategy for sepsis. In the current study, we developed an engineered macrophage releasing IL10, and through a noncontact coculture system, demonstrated that supernatant from engineered macrophages overexpressing IL10 could significantly suppress LPS‐induced endothelial inflammatory response, restore endothelial integrity, and decrease the apoptosis of endothelial cells.

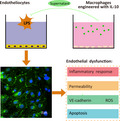

**Abbreviations**
7‐AAD: 7‐aminoactinomycin D
Ccl 7: C‐C Motif Chemokine Ligand 7
CTL: control
DAPI: 4',6‐diamidino‐2‐phenylindole
DCFDA: 2’,7’ –dichlorofluorescin diacetate
DMEM: Dulbecco's Modified Eagle Medium
ECs: endothelial cells
EV: extracellular vesicle
IL10: interleukin 10
IL10‐eM: engineered macrophages overexpression IL10
LPS: lipopolysaccharide
M‐Vec: engineered macrophages not express IL10
M‐Wt: wild‐type macrophages
PVDF: polyvinylidene difluoride
qPCR: quantitative polymerase chain reaction
ROS: reactive oxygen species
SDS/PAGE: sodium dodecyl sulfate/polyacrylamide electrophoresis gels
TEER: electrical resistance
TNFα: tumor necrosis factor α
VEC: vascular endothelial cells

Sepsis is one type of life‐threatening organ dysfunction that is caused by the imbalance of host response to infection and is the most common cause of death for critically ill patients [1, 2]. A main pathophysiological change in sepsis is the dysfunction of vascular endothelial cells (VEC) [3, 4]. Activation and injury of VEC can lead to pro‐coagulant, pro‐inflammatory, and high vascular permeability and eventually lead to multiple organ dysfunction [5, 6, 7]. Therefore, restoration of VEC function is the primary therapeutic strategy for the treatment of sepsis.

IL10 is an anti‐inflammatory cytokine produced by various cells, including T cells, monocytes, macrophages, dendritic cells, and endothelial cells [8]. It is now recognized that IL10 could reduce inflammation by antioxidant properties and inhibit the production of inflammatory factors [9]. In addition, it has been suggested that IL10 played an important role in inhibiting endothelial dysfunction in endotoxemia induced by lipopolysaccharide (LPS) [10]. However, as the disadvantage of all protein drugs, the short half‐life of IL10 *in vivo* hampers the application of disease treatment. Emerging evidence has suggested that macrophage‐based cell therapy and drug delivery are excellent alternative treatments for a variety of diseases [11, 12]. Thus, sustained expression of IL10, delivered by engineered macrophages with a genetic modification approach, may be an effective way to solve this disadvantage.

Macrophages are one of the most important immune cells in the innate immune system and have been suggested to be greatly involved in the microvasculature dysfunction in sepsis. The previous study has highlighted the interconnection between macrophages and endothelial cells in various pathological conditions at the level of growth factor and cytokine signaling [13]. In a noncontact coculture system, macrophages isolated from human blood could enhance the barrier function of endothelial cells [14]. Exosomes released from human macrophages could inhibit endothelial cell migration [15]. Both M2 macrophages and supernatant can increase the viability of endothelial cells (ECs) and facilitate the proliferation of pulmonary ECs in sepsis‐induced acute lung injury [16]. In addition, it was reported that the transplantation of engineered macrophages protected mice from sepsis [17]. Significantly, a landmark study found that bone marrow mesenchymal stem cells can reduce sepsis mortality and improve organ function by reprogramming macrophages to express large amounts of IL10 [18]. However, the effects of macrophages with high expression of IL10 on endothelial dysfunction were not fully understood. Whether genetically modified macrophages releasing IL10 could restore LPS‐induced endothelial integrity and function remains to be explored.

Therefore, the present study aimed to investigate the effect of supernatant from engineered macrophages overexpressing IL10 (IL10‐eM) on the injury and dysfunction of VEC induced by LPS via a noncontact indirect coculture system. In addition, we also wanted to develop an engineered macrophage in this study and provide a basis for the next step of exosome‐based IL10 delivery in sepsis treatment.

## Materials and methods

### Vector constructs and lentivirus transfection

The construction of pCDH vectors and the lentivirus transfection were similar to a previous study [19]. Mouse IL10 cDNA sequence was obtained from the National Center for Biotechnology Information website. IL10 cDNA was cloned into the pCDH‐MSCV‐MCS‐EF1α‐copGFP‐T2A‐Puro (SBI) lentivirus expression vector. 293T cells were transfected with the lentiviral vectors and the packing plasmids, pVSVg and psPAX2, and the viral particles were used to infect RAW264.7 cells. Cells were selected with 10 μg·mL^−1^ puromycin for 2 days. Engineered RAW264.7 cells genetically modified with IL‐10 were marked as IL10‐eM, with an empty vector (not express IL10) were marked as M‐Vec, and wild‐type cells not transfected with lentivirus were marked as M‐Wt.

### Analysis of macrophage polarization by flow cytometry

The polarization of IL10‐eM and M‐Vec was determined by flow cytometry, using antibodies including CD206‐PE (M2), Arg‐1‐APC (M2), and iNos‐PE(M1) (eBioscience, USA) to examine cell surface antigen and characteristic gene of activated M1 and M2 macrophages. The fixation and permeabilization of macrophages were performed according to the manufacturer's protocols (BD, USA). After that, cells were blocked with 1% BSA for 20 min and stained with antibodies for 30 min under the condition of avoiding light. Finally, 20 000 events per test were collected and analyzed with the diva8.0 software (BD Canto II, USA).

### Cell culture and CCK‐8 assay

C166, a mouse vascular endothelial cell line used in this study, was purchased from Honsun Biological Technology Co., Ltd. (Shanghai, China). RAW264.7 cell line was provided by Professor Zhang Luo. C166 cells and RAW264.7 cells were cultured with DMEM (Hyclone^TM^, USA) containing 10%FBS (Sigma, USA) and 1% antibiotics (penicillin and streptomycin; Biosharp, China) at 37 °C in a 5%CO_2_ incubator.

CCK‐8 assay was used to determine endothelium viability (MCE, USA). Briefly, C166 cells were seeded in a 96‐well plate at 5 × 10^3^ cells·well^−1^ and treated with LPS at different doses and time points. Then CCK‐8 was added into the culture medium for 3 h and the absorbance at 450 nm was determined by a microplate reader. Cell viability was calculated according to the manufacturer's protocols.

### Cell noncontact coculture

Macrophages from M‐Wt, M‐Vec, and IL10‐eM groups were cultured with a culture medium containing FBS in 10 cm dishes, respectively. When reached 80–90% confluency, the culture medium was replaced with Dulbecco's modified Eagle medium (DMEM) without FBS and antibiotics and cells continued to be cultured for 24 h. Thereafter, the supernatants from different groups were collected and frozen at −80 °C after centrifuging at 1000 ***g*** for 10 min. In a noncontact coculture system, C166 was treated with LPS to simulate septic endothelial injury, and then, supernatants from different macrophages were added to investigate the effect on endothelial dysfunction. The detailed treatment methods and experimental group settings are shown as below: (a) Control (CTL) group: DMEM cultured for 24 h, (b) LPS group: DMEM containing 1 μg·mL^−1^ LPS (Sigma, USA) was treated for 24 h, (c) M‐Wt + LPS group: M‐Wt supernatant was pretreated for 30 min and then added 1 μg·mL^−1^ LPS for 24 h, (d) M‐Vec + LPS group: M‐Vec supernatant was pretreated for 30 min and then added 1 μg·mL^−1^ LPS for 24 h, (e) IL10‐eM + LPS group: IL10‐eM supernatant was pretreated for 30 min and then added 1 μg·mL^−1^ LPS for 24 h.

### Real‐time qPCR analysis and PCR array analysis

Total RNA was extracted from cells with TRIzol Reagent (Invitrogen, USA) according to the manufacturer's protocols. cDNA was synthesized using a Genomic reverse transcription kit (TOYOBO, Japan), and real‐time PCR was performed with QuantStudio 5 (Applied Biosystems, USA) using 2×SYBR Green qPCR Master Mix (Biomake, USA). All samples were run at least three independent experiments, and the relative gene expression was normalized to internal control cyclophilin. The expression level of each target gene was displayed by the ratio of the CTL group by Quant‐Studio Design and Analysis desktop software (Applied Biosystems, USA). The sequence of primers used for qPCR will be provided under request.

Quantitative polymerase chain reaction (qPCR) arrays are performed to analyze a panel of cytokine‐ and chemokine‐related gene expression in IL10‐eM and M‐Vec macrophages following the instructions of the manufacturer (Wcgene Biotechnology Corporation, China). The results were analyzed using software from Wcgene Biotech.

### ELISA measurement

The supernatant of macrophages from M‐Wt, M‐Vec, and IL10‐eM was collected to detect IL10 levels, and supernatant of VECs from different groups (CTL, LPS, M‐Wt + LPS, M‐Vec + LPS, IL10‐eM + LPS) was used to determine the level of tumor necrosis factor α (TNFα) and IL6 by enzyme‐linked immunosorbent assay (ELISA) kit (Boster, China). All ELISA experiments were performed strictly following the manufacturer's protocols.

### Measurement of permeability in monolayer endothelial cells with TEER and FITC‐Dextran methods

C166 cells were seeded at a density of 5 × 10^4^ cells·well^−1^ in a 24‐well plate Transwells upper chamber with a pore diameter of 0.4 μm (Greiner Bio‐one, Germany). 500 μL and 1500 μL culture medium were added in the upper chamber and the lower chamber, respectively, and the Transwells were cultured for 5–7 days in a 37 °C incubator containing 5%CO2. After the endothelial cells reached full confluence, the medium was aspirated and the upper and lower chambers of Transwells were simultaneously treated with corresponding factors according to different groups. The transendothelial electrical resistance (TEER) value of monolayer endothelial cells was measured by using Millicell ERS‐2 volt ohmmeter (Millipore, USA) according to user guide documents. Subsequently, the 40 kDa‐FITC‐Dextran (Chondrex, Inc. China) was added to the upper chamber (5 mg·mL^−1^) and the 600 μL medium was collected from the lower chamber after 30 min, then fluorescence intensity of 40 kDa‐FITC‐Dextran was measured by microplate assays on a 96‐well plate at 200 μL per well according to the manufacturer's protocols. Briefly, the fluorescence intensity was detected at wavelengths of excitation (490 nm) and emission (520 nm). All the results were given according to the ratio to the CTL group.

### Reactive oxygen species (ROS) production assay

The fluorescence intensity of ROS in different groups was measured using a DCFDA/H2DCFDA—Cellular ROS Assay Kit (Abcam, UK) according to the manufacturer's instructions. Briefly, C166 cells were seeded in a 96‐well plate at 2 × 10^4^ cells·well^−1^ and adhered overnight and then stained with 2’,7’ –dichlorofluorescin diacetate (DCFDA) solution for 45 min at 37 °C in the dark. After being washed with 1×buffer, the cells were subjected to different treatments (CTL, LPS, M‐Wt + LPS, M‐Vec + LPS, IL10‐eM + LPS) and the fluorescence values were detected at different time points with microplate at wavelengths of excitation (485 nm) and emission (535 nm).

### Immunofluorescence staining

Immunofluorescence analysis of VE‐cadherin was performed on C166 endothelial cells. The cells (5 × 10^4^ cells·well^−1^) were grown on a glass coverslip in a 24‐well plate. When reached 80–90% confluent, the cells were subjected to different treatments (CTL, LPS, M‐Wt + LPS, M‐Vec + LPS, IL10‐eM + LPS) for 24 h. After being fixed with 4% paraformaldehyde for 10 min, the cells were blocked with normal goat serum for 30 min at room temperature and then incubated with VE‐cadherin antibody (1 : 50, Boster, China) at 37 °C for 1 h. After the cells were rinsed with PBS, FITC‐conjugated goat anti‐mouse secondary antibodies were added at 37 °C for 1 h, and the cells were subsequently stained with 4′,6‐diamidino‐2‐phenylindole (DAPI, Sigma, USA) for 5 min. Finally, the image of cells was acquired with a confocal laser scanner microscope (Nikon, Japan). The fluorescence intensity of VE‐cadherin was quantified with imagej software.

### Analysis of C166 apoptosis by flow cytometry

Apoptosis of C166 was evaluated with flow cytometry (BD Biosciences) using 7‐AAD/Annexin V PE apoptosis kit (BD Biosciences, USA) according to the manufacturer's instructions. C166 cells (2 × 10^5^ cells·well^−1^) were seeded in 6cm dishes. After growing to 80–90% confluence, the medium was aspirated and different treatments were added for 24 h. Then, the cells were digested and rinsed with PBS. After centrifugation, cells of each group were resuspended with 1× binding buffer at a concentration of 1 × 10^6^ cells·mL^−1^, and then, 100 µL of the solution (1 × 10^5^ cells) was transferred to a flow cytometric tube. Five µL of Annexin V PE and 5 µL of 7‐aminoactinomycin D (7‐AAD) were added to the tube and incubated for 15 min according to the instructions. Then, the cells were analyzed with flow cytometry within 1 h. For each sample, 20 000 events were collected and analyzed with the cellquest software (BD FACSCalibur).

### Western blot analysis

Proteins were lysed from C166 in 6‐well plates using RIPA lysis buffer containing protease inhibitors (Boster Biological Technology co. Ltd, China). After measuring the protein concentration with the BCA protein assay kit (Boster Biological Technology co. Ltd, China), proteins from each sample with the same quality (15 μg) were separated on sodium dodecyl sulfate/polyacrylamide electrophoresis gels (SDS/PAGE) and transferred to polyvinylidene difluoride (PVDF) membranes (Millipore). Membranes were blocked with 5% milk resolved in PBST buffer and probed with diluted antibodies. Primary antibodies were Ve‐cadherin (Boster Biological Technology co. Ltd, China) and GAPDH (Proteintech Group Inc., USA).

### Statistical analysis

Data were analyzed with the statistical software spss19.0 and graphpad Prism 8.0, and the data were expressed by mean ± SD (standard deviation). Two‐tailed paired‐samples *t*‐test was used to evaluate the differences between groups. *P* < 0.05 was considered statistically significant. The error bars in the all figure indicate SD.

## Results

### Characterization of engineered macrophages overexpressing IL‐10

**Fig. 1:**
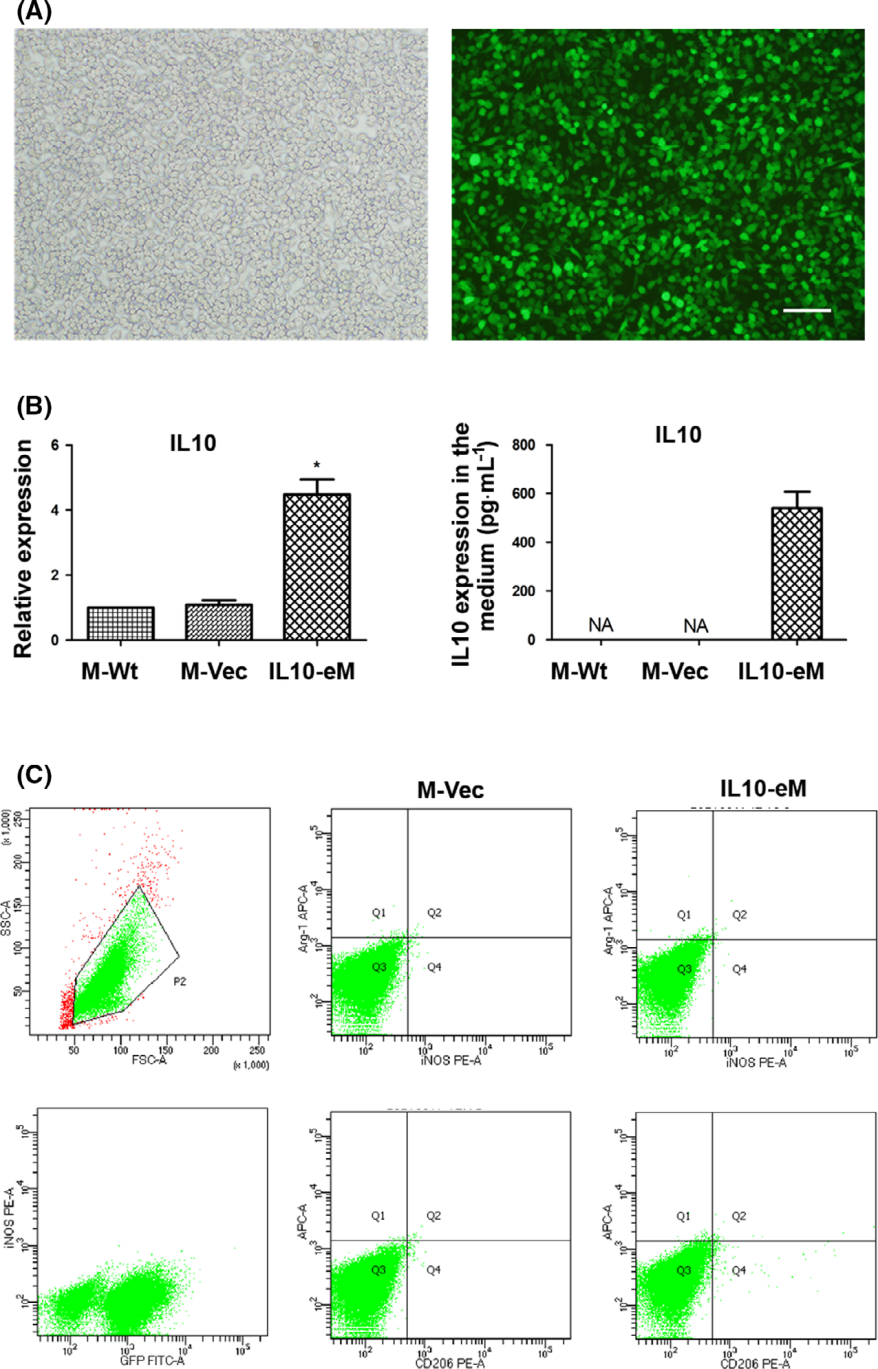
Establishment and characterization of engineered macrophage with releasing IL10 (IL10‐eM). (A) The morphology of RAW264.7 cells overexpressing IL10 under optics (Left) and fluorescence microscope (Right). Bar = 200 μm. (B) Expression of IL10 was determined by real‐time qPCR (left) and ELISA (right), NA means ‘not available’. (*n* = 3, *P < 0.05 vs M‐Wt) (C) The polarization of IL10‐eM and M‐Vec were determined by flow cytometry. CD206 was an M2 cell surface marker. Arg‐1‐APC and iNos‐PE were the characteristic genes of activated M2 and M1 macrophages, respectively (*n* = 2). The error bars indicate SD and two‐tailed paired‐samples *t*‐test was used to evaluate the differences between groups.

RAW264.7 macrophages genetically modified with IL10 (IL10‐eM) were constructed using lentiviral vectors (Fig. 1A). The expression of IL10 in the IL10‐eM cells was detected by real‐time PCR and ELISA, respectively. Higher expression of IL10 mRNA was observed in IL10‐eM and more than 500 pg·mL^−1^ IL10 was detected in the supernatant from IL10‐eM after cultured 24 h (Fig. 1B). The polarization of IL10‐eM and M‐Vec was determined by flow cytometry. Both IL10‐eM and M‐Vec macrophages are negative for iNOS, Arg‐1, and CD206, suggesting that high expression of IL‐10 did not induce engineered macrophages toward M1 or M2 phenotypes (Fig. 1C). PCR array was used to determine whether macrophages modified with IL10 would influence cytokine and chemokine expression. The results showed that high expression of IL10 induces downregulation of C‐C Motif Chemokine Ligand 7(Ccl7), Ccl14, IL1β, and upregulation of Ccl19, and heatmap is presented in Fig. S1. In addition, we found that LPS stimulation significantly blocked the expression of IL10 in IL10‐eM (Fig. S2).

### Supernatant from engineered macrophages mitigated the LPS‐induced inflammatory response of VECs

**Fig. 2:**
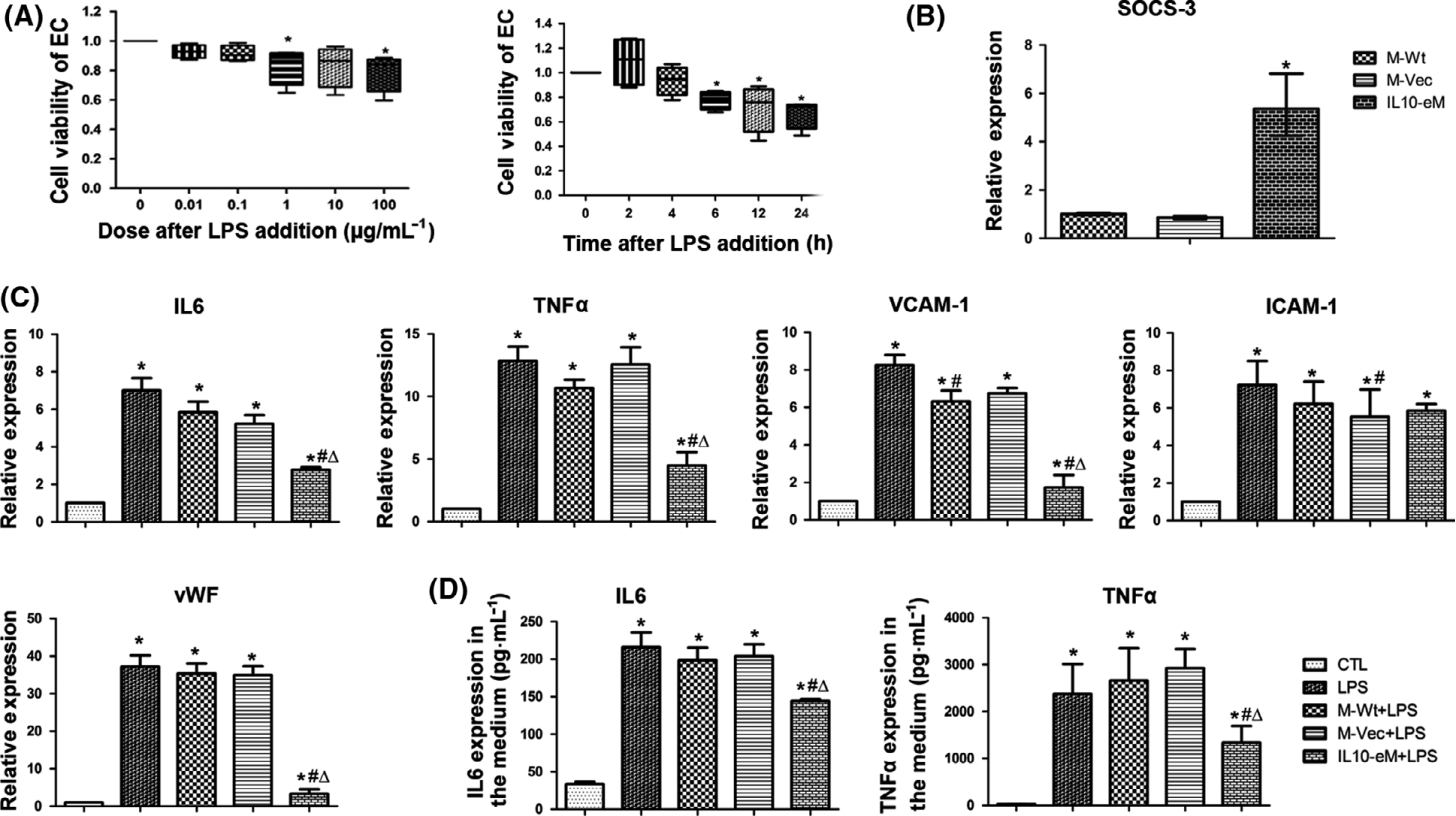
IL10‐eM supernatant protected against LPS‐induced VECs activation and injury. (A) Cell viability of C166 under different doses of LPS (left) and different time points at 1 μg·mL^−1^ LPS (right). (*n* = 4) (B) Quantification of SOSC‐3 expression on endothelium following supernatant treatment. (*n* = 3) *P < 0.05 vs M‐Wt, (C) Expression of inflammatory mediators (IL6, TNFα), endothelial cell activation markers (VCAM‐1, ICAM‐1), and endothelial cell injury marker (vWF) were detected by real‐time qPCR. (*n* = 3) (D) Expression of IL6 and TNFα in cell supernatant determined by ELISA. (*n* = 3) *P < 0.05 vs CTL, #*P* < 0.05 vs LPS, Δp < 0.05 vs M‐Wt + LPS. The error bars indicate SD and two‐tailed paired‐samples *t*‐test was used to evaluate the differences between groups.

To simulate vascular endothelial injury in sepsis, we explored the timing and dose of LPS by determining the viability of VECs (Fig. 2A). Based on the CCK‐8 results, VECs were treated with 1 μg·mL^−1^ LPS for 24 h to recapitulate a sepsis environment in our study. To confirm the activation of IL‐10 on endothelium, we determined the expression of SOCS‐3, a downstream gene of IL10 reported previously [20]. Supernatant from macrophage overexpressing IL10 significantly increased SOCS‐3 expression (Fig. 2B). IL6 and TNFα were the most common inflammatory mediators. Real‐time PCR showed that the mRNA of IL6 and TNFα was significantly increased upon LPS challenge and the presence of IL10‐eM supernatant could significantly inhibit LPS‐induced elevation of IL6 and TNFα (Fig. 2C). Remarkably increased expression of VCAM‐1 and ICAM‐1, which were endothelial cell activation markers, was observed in the LPS‐treated endothelial cells. However, the cotreatment of IL10‐eM supernatant could abrogate the effects of LPS on VCAM‐1 and endothelial injury marker vWF (Fig. 2C). ELISA was performed to determine the expression of IL6 and TNFα in the coculture medium. In agreement with the real‐time PCR results, the presence of IL10‐eM supernatant could significantly mitigate LPS‐induced upregulation of IL6 and TNFα (Fig. 2D).

### IL10‐eM supernatant decreased endothelial permeability induced by LPS

**Fig. 3:**
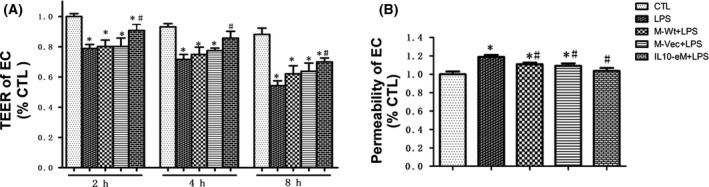
IL10‐eM supernatant could partially restore the integrity of the endothelial cell monolayer. (A) LPS challenge decreased the TEER value, and treatment with IL10‐eM supernatant could antagonize LPS‐induced downregulation of TEER at different time points (2 h, 4 h, 8 h). (*n* = 5) (B) Permeability of VEC was determined by the leakage rate of 40KD dextran‐FITC. (*n* = 6) *P < 0.05 vs CTL, #*P* < 0.05 vs LPS. The error bars indicate SD and two‐tailed paired‐samples *t*‐test was used to evaluate the differences between groups.

The endothelial cell monolayer integrity was evaluated by the permeability of endothelial cell monolayer using transendothelial electrical resistance (TEER) and the leakage rate of 40 KD dextran‐FITC. LPS treatment could significantly reduce the TEER value of the endothelial cell monolayer at different time points and increase the leakage rate of dextran‐FITC (Fig. 3). Notably, the presence of supernatant from M‐Wt or M‐Vec increased the TEER value, although there was no statistically significant difference (Fig. 3A). The leakage of 40 KD dextran‐FITC was also considerably inhibited by the cotreatment with M‐Wt or M‐Vec supernatants (Fig. 3B). Significantly, treatment with IL10‐eM supernatant could antagonize the LPS‐induced downregulation of TEER value and upregulation of the leakage rate of dextran‐FITC.

### IL10‐eM supernatant attenuated the LPS‐induced VE‐cadherin downregulation and ROS generation

**Fig. 4:**
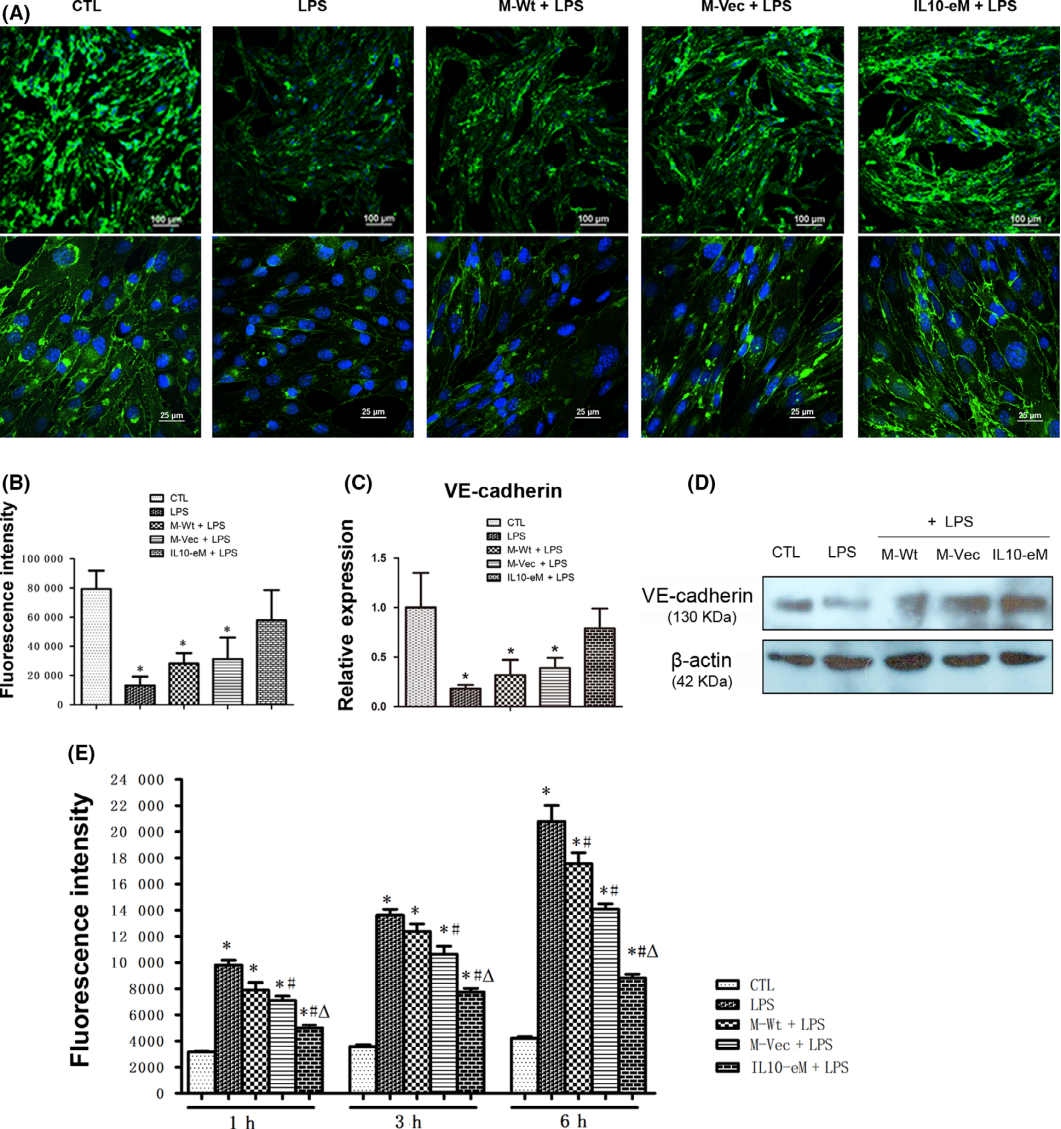
IL10‐eM supernatant inhibited LPS‐induced downregulation of VE‐cadherin and ROS generation. (A) The image of VE‐cadherin was detected with confocal scanning at lower magnification (top, bar = 100 μm). High‐quality fluorescence image (bottom, bar = 25 μm). (*n* = 3; five visual fields were taken randomly from each sample for analysis). (B) Quantification analysis of fluorescence intensity of VE‐cadherin. (*n* = 3). (C) Quantification of VE‐cadherin by real‐time PCR. (*n* = 3). (D) Western blot results of VE‐cadherin. (*n* = 3) (E) ROS production was detected with ROS Assay Kit at different time points (1 h, 3 h, 6 h). (*n* = 5) *P < 0.05 vs CTL, #*P* < 0.05 vs LPS, Δp < 0.05 vs M‐Wt + LPS. The error bars indicate SD and two‐tailed paired‐samples *t*‐test was used to evaluate the differences between groups.

VE‐cadherin is a major molecule of endothelial cell junction. The expression of VE‐cadherin was determined with immunofluorescence and real‐time PCR (Fig. 4A,C). Quantification of VE‐cadherin expression by qPCR and immunofluorescence staining showed that cotreatment with IL10‐eM supernatant could greatly suppress the effects of LPS and restore the expression of VE‐cadherin similar to the level of the control group (Fig. 4B,C). The expression of VE‐cadherin was also confirmed by western blot at protein level (Fig. 4D).

Reactive oxygen species (ROS) was considered to be one of the major mechanisms of endothelial cell injury in sepsis. The production of ROS in all groups of VECs was determined with ROS Assay Kit. Fluorescence intensity of ROS significantly increased in the LPS group, while IL10‐eM supernatant considerably alleviated LPS‐induced generation of ROS (Fig. 4E). Notably, cotreatment with supernatants from M‐WT or M‐VEC also partially reduced LPS‐induced ROS production (Fig. 4E).

### IL10‐eM supernatant ameliorated apoptosis of VEC induced by LPS

**Fig. 5:**
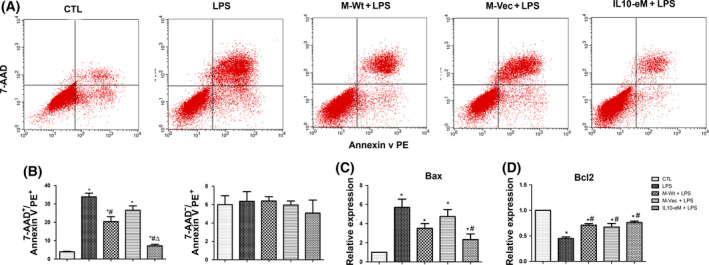
IL10‐eM supernatant suppressed the LPS‐induced apoptosis of VECs. (A) Flow cytometry was used to detect endothelial cell apoptosis. (B) LPS remarkably increased the percentage of 7‐AAD^+^/Annexin V PE^+^ cells, and treatment with IL10‐eM supernatant significantly mitigated the effect of LPS. (*n* = 3) (C,D) The expression levels of Bax and Bcl2 were detected using real‐time qPCR. (*n* = 3). *P < 0.05 vs CTL, #*P* < 0.05 vs LPS, Δp < 0.05 vs M‐Wt + LPS. The error bars indicate SD and two‐tailed paired‐samples *t*‐test was used to evaluate the differences between groups.

The protective effect of IL10‐eM supernatant against LPS‐induced apoptosis of VECs was determined by flow cytometry using 7‐AAD/Annexin V PE kit. The percentage of 7‐AAD^+^/ Annexin V PE^+^ apoptotic endothelial cells was significantly increased in the LPS group (33.8%), but IL10‐eM supernatant could reduce the percentage of apoptosis to 7.23% (Fig. 5A,B). Decreased apoptosis was also observed in the presence of M‐Wt supernatant (Fig. 5B). However, no difference in 7‐AAD^‐^/Annexin V PE^+^ was observed among all groups (Fig. 5A,B). At the molecular level, it was found that cotreatment with IL10‐eM supernatant could significantly attenuate LPS‐induced upregulation of Bax and downregulation of Bcl2(Fig. 5C,D). Notably, the cotreatment with supernatant from M‐Wt or M‐Vec also significantly antagonized the effects of LPS on Bcl2 expression (Fig. 5D).

## Discussion

Previous studies suggested that IL10 could inhibit the release of inflammatory mediators, reduce the adhesion of lymphocytes, platelets, and other blood cells to endothelial cells, and decrease the ECs apoptosis [21, 22, 23, 24]. In addition, the LPS‐induced expression of pro‐inflammatory cytokines (such as TNFα, IL6, and IL1) also can be inhibited by IL10 [18, 21]. Our results showed that IL10‐eM supernatant, containing a high level of IL10, led to the release of lower amounts of TNF‐α and IL‐6 in endothelial cells, suggesting that IL10‐eM supernatant could attenuate the inflammatory response in LPS‐treated ECs.

Endothelial cell injury and increased permeability are typical pathological changes during sepsis [3, 4]. Microvascular barrier breakdown is a very important feature of endothelial cell dysfunction in sepsis [25]. Previous studies showed that IL10 decreased the permeability of BBB in the severe acute pancreatitis model and prevented microvascular protein leakage [23, 24]. In this study, with the help of the LPS‐induced endothelial cell injury model and noncontact coculture system, we found that the supernatant of IL10‐eM mitigated the LPS‐induced increase of endothelial cell permeability.

Endothelial cell permeability is regulated by many factors, mainly involving intercellular junctions. VE‐Cadherin is a primary adhesion junction molecule and plays an important role in modulating endothelial permeability [26]. It was reported that ROS may participate in the regulation of endothelial barrier function during LPS stimulation [26]. Consistently, our study found that IL10‐eM supernatant significantly alleviated the downregulation of VE‐Cadherin and high ROS production upon the LPS challenge. These changes may greatly contribute to the protective effects of supernatant of IL10‐eM on the barrier function of endothelial cells.

Our study showed that the presence of IL10‐eM supernatant could significantly alleviate the effect of LPS on apoptosis of the endothelial cell. At the molecular level, LPS‐induced upregulation of Bax and downregulation of Bcl2 were significantly reversed by the presence of IL10‐eM supernatant. Supportively, many studies have shown that IL10 significantly inhibited the apoptosis of endothelial cells caused by various factors [24, 27, 28]. Interestingly, our results suggested that supernatants from wild‐type macrophages also partially inhibited LPS‐induced endothelial apoptosis.

It is worth noting that treatment with supernatants from M‐Wt or M‐Vec also has some beneficial effects against LPS‐induced endothelial injury and dysfunction. The leakage rate was significantly mitigated by cotreatment with M‐Wt or M‐Vec supernatants. In addition, the presence of M‐Wt significantly inhibited endothelial cell apoptosis induced by LPS. Besides, the presence of M‐Wt or M‐Vec supernatants partly reduced LPS‐induced ROS production and increased the expression of VE‐cadherin. The underlying mechanism we proposed is that extracellular vesicles carrying miRNA and cytokines secreted by macrophages in supernatant probably conferred endothelial cells against LPS‐induced dysfunction and injury to a certain extent. Consistently, it was reported that macrophages contributed to the enhanced barrier function of endothelial cells [14, 29]. Exosomes generated from perivascular macrophages could influence endothelial behavior [30]. It is worth noting that, extracellular vesicle‐encapsulated IL10 (IL10 + EVs) derived from engineered macrophages could protect against ischemic acute kidney injury effectively [31]. Therefore, we proposed that IL10 in the supernatant of IL10‐eM was primarily responsible for protective effects against LPS‐induced endothelial dysfunction; however, exosome and cytokine secreted by macrophage may also play an important role in this process.

The current study provided *in vitro* evidence that supernatant from IL10‐eM protected endothelial cells against LPS‐induced injury and dysfunction. However, our study also had some limitations. Sepsis is a very complex disease, and LPS treatment can not truly recapitulate vascular injury and dysfunction in the septic microenvironment. This study is only a *in vitro* cellular experiment and further animal model evidence is needed to test whether the supernatant of IL10‐eM can protect the vascular injury and prevent leakage in sepsis. In addition, we found that IL10 production of IL10‐eM under LPS stimuli was significantly suppressed, which indicated that the transplantation of IL10‐eM in septic models may be ineffective. Whether extracellular vesicle‐encapsulated IL‐10 isolated from IL10‐eM supernatant could serve as nanotherapeutics against sepsis is what we are going to do in the follow‐up study.

In conclusion, this study developed an engineered macrophage releasing IL10 and demonstrated that supernatant from engineered macrophages overexpressing IL10 could significantly inhibit LPS‐induced endothelial inflammatory response, restore endothelial integrity, and decrease the apoptosis of endothelial cells via a noncontact coculture system.

## Conflict of interest

The authors declare that they have no known competing financial interests or personal relationships that could have appeared to influence the work reported in this paper.

## Author contributions

LXY and TJW designed and implemented the experiments and wrote the manuscript. PHN, L Zhu, MMG, and SHY also conducted some experiments. HXJ, XBL, and L Zhang, PL M analyzed the experimented data. YX, PL M, and MH reviewed and revised the manuscript.

## Supporting information


**Fig. S1**. Heatmap of cytokine/chemokine PCR array results between IL10‐eM and M‐Vec.

**Fig. S2**. Expression of IL10 in supernatant from IL10‐eM and M‐Vec with and without LPS treatment by ELISA analysis. LPS stimulation significantly suppressed the expression of IL10 in IL10‐eM cells.

## Data Availability

All data in our study are available from the corresponding author on reasonable request.
