# Supplementary figures and images for: Overexpression of interleukin‐10 in engineered macrophages protects endothelial cells against LPS‐induced injury in vitro
Source: FEBS Open Bio. 2022 Jan 26;12(3):605–15. doi: 10.1002/2211-5463.13365 (PMC8886523; doi:10.1002/2211-5463.13365)

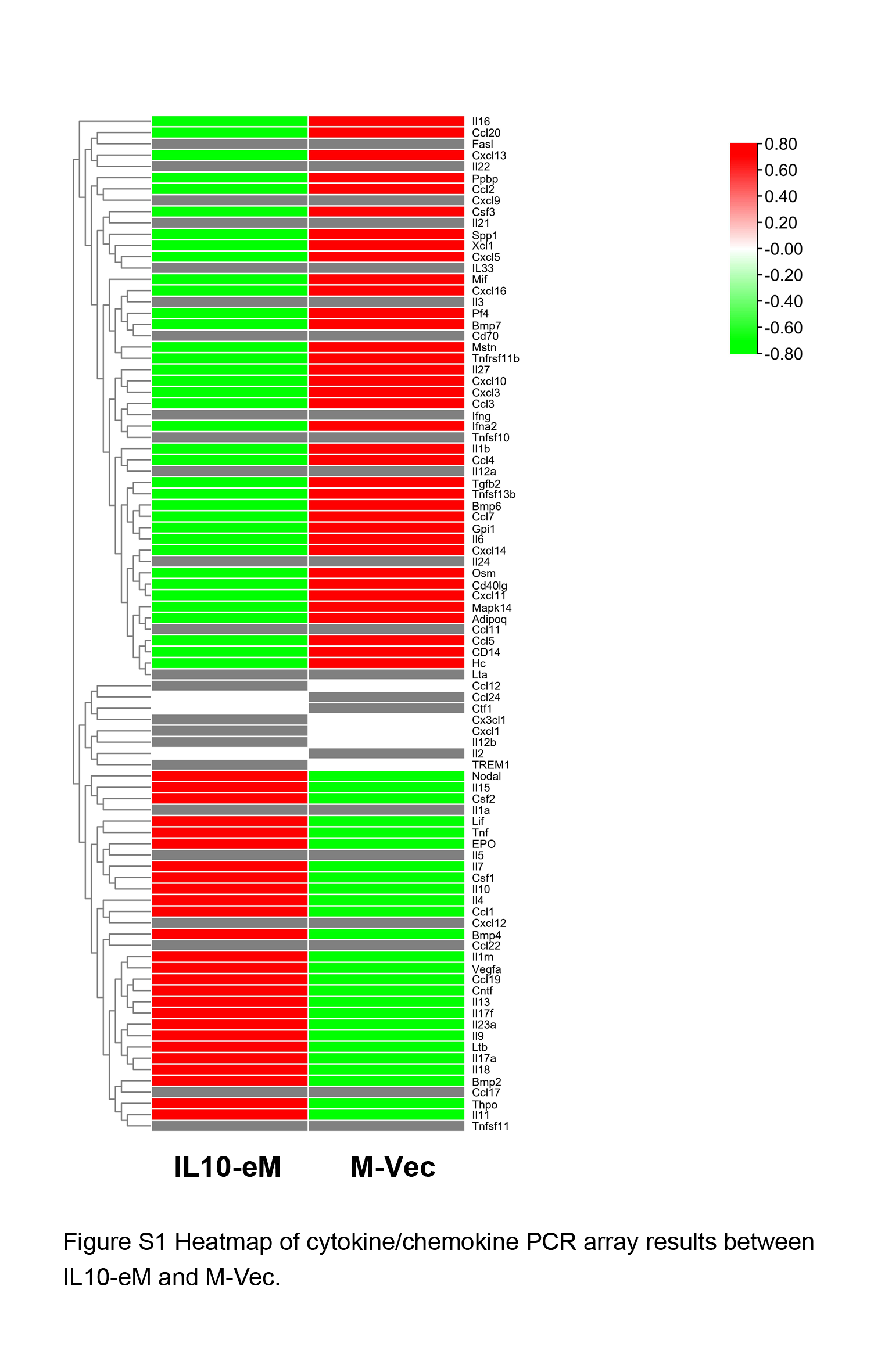

Supplement: Supplementary file 1 — Fig. S1. Heatmap of cytokine/chemokine PCR array results between IL10‐eM and M‐Vec. [file FEB4-12-605-s002.tif]

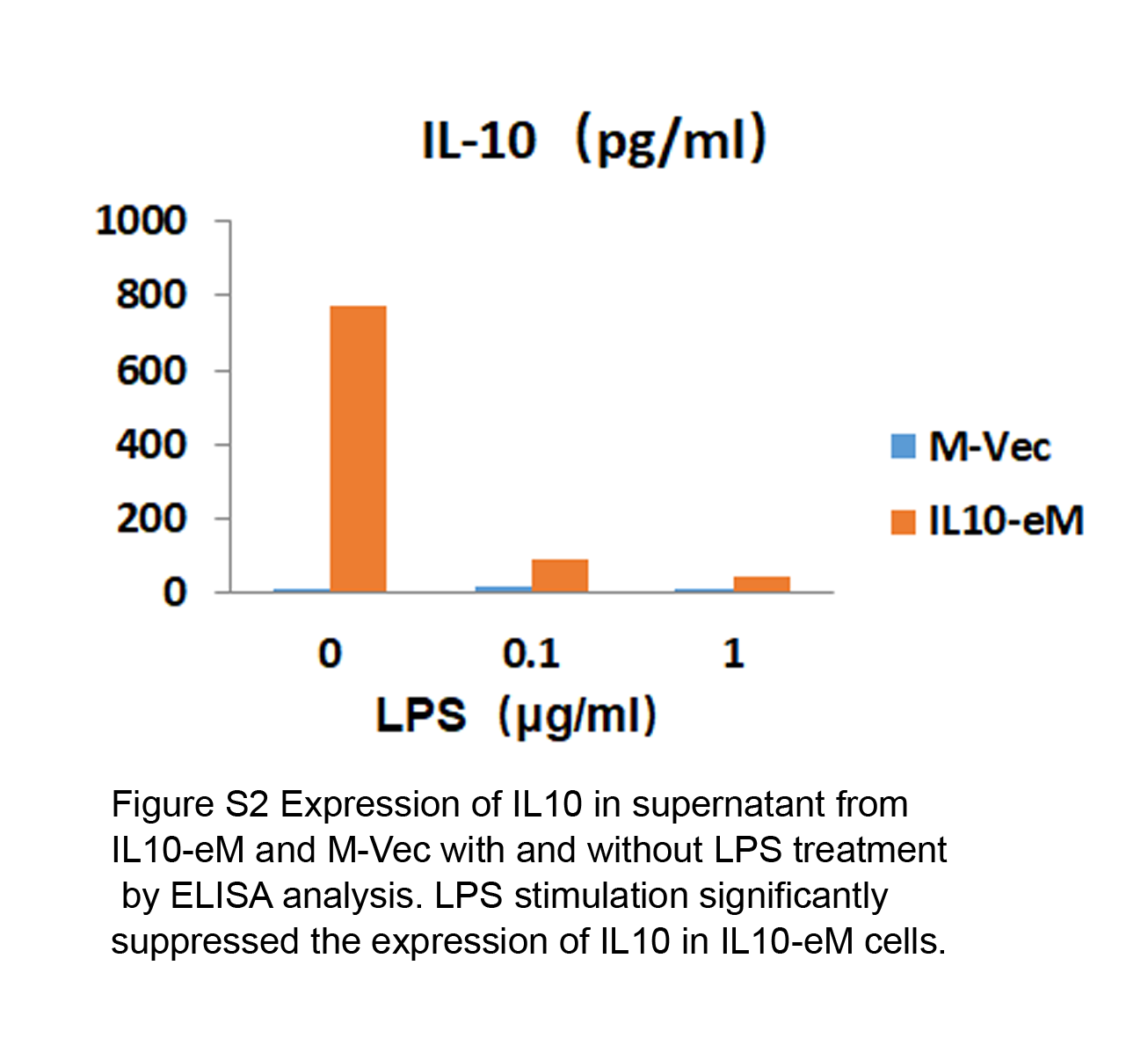

Supplement: Supplementary file 2 — Fig. S2. Expression of IL10 in supernatant from IL10‐eM and M‐Vec with and without LPS treatment by ELISA analysis. LPS stimulation significantly suppressed the expression of IL10 in IL10‐eM cells. [file FEB4-12-605-s001.tif]
